# DUB-independent regulation of pVHL by OTUD6B suppresses hepatocellular carcinoma

**DOI:** 10.1007/s13238-020-00721-x

**Published:** 2020-04-22

**Authors:** Xiaoming Dai, Jing Liu, Wenyi Wei

**Affiliations:** Department of Pathology, Beth Israel Deaconess Medical Center, Harvard Medical School, Boston, MA 02115 USA

Oxygen is vital for most living organisms. During the course of evolution, animals have developed a highly conserved and elegant pathway to regulate oxygen sensing that converges on the heterodimeric transcription factor called hypoxia-inducible factor (HIF), which contains HIF-1α, a labile alpha subunit and HIF-1β, a stable beta subunit (Wang et al., 1995; Kaelin & Ratcliffe, 2008). In the presence of oxygen, HIF-1α is hydroxylated on the proline-402 and proline-564 residues by the family of Egg-Laying Defective Nine dioxygenases (EglN), which are also called Prolyl Hydroxylase Domain (PHD) proteins (Bruick & McKnight, 2001; Epstein et al., 2001; Ivan et al., 2002). The proline hydroxylation post-translational modification subsequently recruits the Cullin 2^VHL^ E3 ubiquitin ligase complex which comprises the von Hippel-Lindau tumor suppressor (pVHL), Elongin B, Elongin C, Rbx1 and Cullin 2 (Zhang et al., 2019). Specific recognition of the proline-hydroxylation modification by Cullin 2^VHL^ leads to the ubiquitination and subsequent proteasomal degradation of HIF-1α. As such, under low oxygen conditions, deficit in the proline hydroxylation of HIF-1α would lead to its stabilization and activation, thus promoting the transcription of hundreds of target genes, such as vascular endothelial growth factor and erythropoietin (Zhang et al., 2019). These HIF-1α target genes normally serve to promote acute or chronic adaptation to hypoxia, facilitate angiogenesis and thus favor the growth of solid tumor (Wilson & Hay, 2011).

pVHL functions largely as a tumor suppressor and germ line mutations in the *VHL* gene cause von Hippel–Lindau disease, a hereditary neoplastic disease associated with clear-cell renal-cell carcinomas (ccRCCs) (Gossage et al., 2015). Disruption of *VHL*, by somatic mutation, hypermethylation of its promoter or chromosomal deletion, is the most recurrent mutation in sporadic ccRCC (Gossage et al., 2015). However, the function and regulation of pVHL in other cancer types such as hepatocellular carcinoma (HCC) remains largely elusive.

Moreover, although pVHL dictates the ubiquitination and degradation of HIF-1α, pVHL itself is also unstable and actively undergoes ubiquitination and degradation. Several ubiquitinating enzymes such as E2-EPF ubiquitin carrier protein (UCP) (Jung et al., 2006) and WD repeat and SOCS box-containing protein 1 (WSB1) (Kim et al., 2015) have been previously reported to regulate pVHL protein stability through promoting its ubiquitination and degradation. Although the VDU1 deubiquitinase (DUB) has been reported to interact with pVHL, it was validated as a pVHL downstream substrate (Li et al., 2002). However, the identity of the physiological DUB that stabilizes pVHL by antagonizing its ubiquitination process remains largely unknown. In a recent remarkable study published in *Advanced Science*, Lingqiang Zhang group reported the ovarian-tumor domain containing protein 6B (OTUD6B) in regulating pVHL protein stability to impact HCC metastasis (Liu et al., 2020).

Liver cancer in which HCC is the major form is the third leading cause of cancer deaths in the world, and more than 50% of HCC patients are in China (Bray et al., 2018). Using siRNA-based targeted screening, the authors found that OTUD6B, but not other OTU family members, could significantly suppress HCC cells migration and metastasis. To explore the underlying molecular mechanism, through RNA-sequencing, they found that HIF-1α-related transcriptional signatures are relatively enriched in *OTUD6B* knockdown cells. As such, depletion of endogenous *OTUD6B* leads to stabilization of HIF-1α, while ectopic over-expression of OTUD6B promotes the ubiquitination of HIF-1α. These results coherently suggest that it may serve as a negative regulator of HIF-1α.

Given the fact that pVHL is the well-characterized upstream negative regulator of HIF-1α, the Zhang group went on to explore the potential regulation of VHL by OTUD6B. Indeed, further biochemical studies showed that, OTUD6B binds directly with pVHL rather than HIF-1α. More importantly, OTUD6B protects pVHL from proteasome dependent degradation via decreasing pVHL Lys 48 ubiquitination, but this function appears to be largely independent of OTUD6B’s enzymatic activity. Moreover, mutant forms of OTUD6B with deletion of OTU domain or mutating the putative catalytic active sites could still suppress the ubiquitination of pVHL, which is consistent with previous report showing that OTUD6B is incapable of cutting any di-ubiquitin *in vitro* (Mevissen et al., 2013). Instead of the OTU catalytic domain, the N-terminal of OTUD6B seems to play a major function in binding and protecting pVHL from degradation. Hence, it is possible that OTUD6B might function as a scaffold to couple pVHL with Elongin B/C to form a stable Cullin 2^VHL^ E3 ligase complex, which protects pVHL from proteasomal degradation. On the other hand, depletion of *OTUD6B* results in the dissociation of Cullin 2^VHL^ complex and the degradation of pVHL presumably by known upstream E3 ligases such as WSB1(Kim et al., 2015) or E2-EPF-UCP (Jung et al., 2006). Consistent with this mechanism, over-expression of pVHL could antagonize *OTUD6B* depletion-induced effects in HCC cell migration and metastasis. However, further investigation is warranted to identify the physiological DUB that can remove the polyubiquitination chain from pVHL to antagonize the functions of the E3 ligases towards pVHL. Given that OTUD6B is integrated into the Cullin 2-Elongin B/C complex, it’s interesting to speculate whether it can form additional E3 complex besides pVHL to play a more general role together with Cullin 2-Elongin B/C. In addition, why does pVHL only bind with OTUD6B but not other OTU family members, such as OTUD6A? To this end, further structural study regarding the difference between the N-terminal domain (NTD) of OTUD6B and OTUD6A could provide more insights. Furthermore, the *in vivo* biological function of OTUD6B remains unclear, and *Otud6b* KO mice will be very helpful to address it in the future studies.

Interestingly, in keeping with an important role of OTUD6B in regulation of pVHL as a critical component of the oxygen sensing pathway, OTUD6B expression was markedly induced under hypoxic condition, suggesting OTUD6B may be a transcriptional target of HIF. Through luciferase reporter assay and chromatin immunoprecipitation assay, the authors found that HIF-1α binds with the promoter of *OTUD6B*. Hence, these data suggest that as a transcriptional target gene of HIF, OTUD6B expression could be induced under the hypoxic condition to stabilize pVHL and promote the degradation of HIF-1α, thus forming a negative feedback loop in regulating HIF activity and oxygen sensing homeostasis.

**Figure 1:**
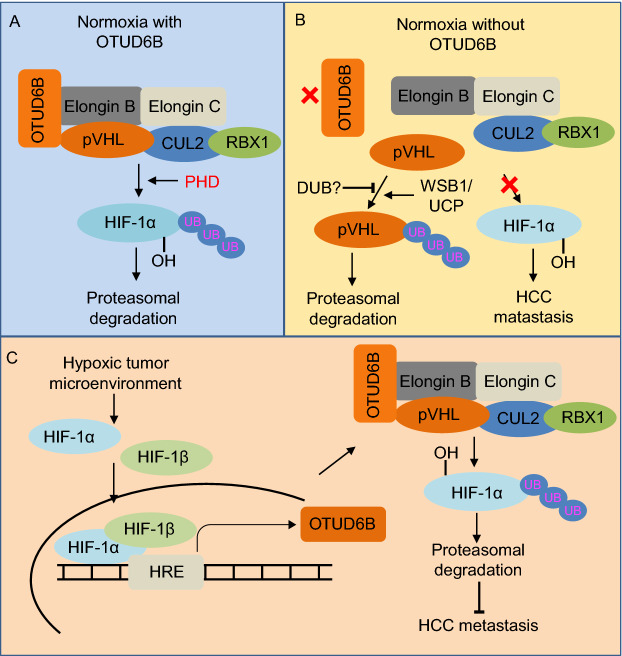
**A schematic representation of pVHL-HIF signaling regulation by OTUD6B**. (A) Under normoxia conditions, OTUD6B functions as a scaffold coupling pVHL and Elongin B/C to form stable Cullin 2^VHL^ E3 ligase complex, promoting HIF-1α proteasomal degradation. (B) Under nomoxia conditions, without OTUD6B, pVHL and Elongin B/C cannot form stable Cullin 2^VHL^ E3 ligase complex, pVHL will be ubiquitinated by WSB1/UCP and degraded. (C) Under hypoxic tumor microenvironment, HIF is activated, increasing the transcription of *OTUD6B*. OTUD6B enhances the formation of stable Cullin 2^VHL^ E3 ligase complex, which protects pVHL from proteasomal degradation. In turn, pVHL suppress HIF activation by targeting it for degradation, thus forming a negative feedback loop to inhibit HCC metastasis

Taken together, this work reveals a new layer of molecular mechanism for the stability regulation of the pVHL tumor suppressor and reveals its potential clinical importance in HCC metastasis and treatment. To this end, OTUD6B was identified through a siRNA screening as a new subunit of the Cullin 2^VHL^ complex, which functions to promote the binding between pVHL and Elongin B/C, thereby protecting the complex from proteasome mediated degradation (Fig. 1). This elegant work therefore adds a new layer for the regulation of oxygen sensing machinery and sheds light on targeting hypoxic microenvironment for HCC therapy.
